# Socio-demographic trends in overweight and obesity among parous and nulliparous women in Ghana

**DOI:** 10.1186/s40608-016-0124-2

**Published:** 2016-11-03

**Authors:** Derek Anamaale Tuoyire, Akwasi Kumi-Kyereme, David Teye Doku

**Affiliations:** 1 Department of Community Medicine, University of Cape Coast, Cape Coast, Ghana; 2 Department of Population & Health, University of Cape Coast, Cape Coast, Ghana; 3 School of Health Sciences, University of Tampere, Tampere, Finland

**Keywords:** Obesity, Overweight, Parous, Nulliparous, Women, Ghana

## Abstract

**Background:**

Overweight and obesity are among the leading threats to global health because of their association with increased risk of morbidity and mortality. Much of the research on overweight and obesity among women largely generalize without due cognisance to differences in their reproductive history. This study explored differences in trends in overweight/obesity, and associated factors between parous and nulliparous women in Ghana.

**Methods:**

Anthropometric measurements from three nationally representative Ghana Demographic and Health Surveys (2003, 2008 and 2014) were analysed using descriptive statistics and multivariate binary logistic regression.

**Results:**

Over all, overweight/obesity increased between 2003 and 2014, with disproportionately higher rates among parous women (from about 30 % in 2003 to about 48 % in 2014) than nulliparous women (from about 15 % in 2003 to about 24 % in 2014). Age, wealth quintile and marital status were associated with overweight/obesity similarly in both women groups. However, there were variations in the association between parous and nulliparious women by educational level, type of locality, occupation and ethnicity.

**Conclusion:**

The trend of overweight/obesity in Ghana warrants urgent national level public health attention to help curb the situation. Such interventions should be tailored bearing in mind the peculiar differences in associated factors between parous and nulliparous women.

## Background

Overweight and obesity are among the leading threats to global health mainly because of their close association with numerous non-communicable chronic diseases and mortality [1]. In 2014, about 2 billion adults, at least 18 years old, were reported to be overweight or obese [1, 2]. Although a global problem, developing countries are currently experiencing alarming increases in prevalence of overweight and obesity compared with developed countries [1, 3]. At the macro level, the rapid upward trend in overweight and obesity in developing countries has largely been attributed to ongoing demographic and nutritional transitions, mainly due to urbanisation and attendant substantial changes in lifestyle; notably reduced physical activity and higher caloric dietary patterns [4, 5]. As a result of these developments, projections are that, over the next two decades, these countries will continue to experience some of the largest proportional increases ranging from 62–205 % and 71–263 % for overweight and obesity, respectively [6]. In sub-Saharan Africa in particular, overweight and obesity are estimated to increase by about 35 % within the next 10 years at a rate of about 5 % per annum [7].

Overweight and obesity vary greatly between men and women, with women across the globe disproportionately affected [3, 8]. Explanations to this disparity mainly refer to general weight gain in women in their childbearing years, gestational weight gain and/or weight retention and adverse lifestyle risk factors associated with pregnancy and the postpartum period [9, 10]. In view of these reproductive factors, the association between parity (number of children born to a woman) and obesity has been intensely investigated. Generally, women with higher parities have been found to have higher retention of gestational weight gain and consequently onset of overweight and obesity [11, 12].

The real impact of parity and associated reproductive factors could, however, be modest and intertwined in a complex pattern with socio-cultural, demographic, and socio-economic factors, as well as other risk factors [13]. Indeed, demographic and socio-economic factors [14, 15] have gained much attention in recent times, largely due to their potential to readily identify at-risk groups for targeted intervention purposes. Overweight and obesity tend to be strongly associated with socioeconomic status, however, the direction of association has been found to vary with levels of economic development [16]. In contrast to high income countries, overweight and obesity have positively been associated with wealthier, more educated and urban dwellers in developing countries [16, 17]. Various studies have similarly reported associations between age [18], marital status [19], ethnicity [20], education [20, 21] and occupation [21] and overweight and obesity.

Despite a significant avalanche of studies on overweight and obesity over the last three decades, little is known about whether the phenomenon and its associated factors operate similarly or dissimilarly between women who have initiated childbearing and those who have not or are yet to. Some previous studies typically adjust for parity to account for the effects of reproductive risk factors, however, this does not sufficiently highlight the extent to which the factors associated with overweight and obesity operate differently or otherwise between women who have initiated childbearing and those who have not or are yet to. In an increasingly more obesogenic and diverse population of women, childbearing may aggravate the risk of overweight and obesity more than previously understood. Alternatively, with higher rates of overweight and obesity among the generality of women, the effects of childbearing on associated risk factors may be less salient than is previously thought. In view of this, we hypothesize that socio-demographic factors associated with overweight and obesity operate in fundamentally different ways between parous (previously given birth) and nulliparous (never given birth) women.

Particularly, for women in developing countries, reproductive factors in concert with cultural valuation of large body size and the nutrition transition could exacerbate their risks of developing overweight and obesity. Hence, the present study focuses on Ghana, which like many other countries in sub-Saharan Africa is faced with exponential rises in the prevalence of overweight and obesity among women. Demographic and Health Surveys (DHS) in Ghana indicate that the percentage of women aged 15–49 years who were overweight or obese increased from 13 % in 1993 to 40 % in 2014 [22, 23]. Dake [24] observed that the prevalence of obesity alone among Ghanaian women in the same age group increased from 3.4 % in 1993 to 9.2 % in 2008, representing close to a three-fold increase over the 15-year period. Based on the calculated rate of increase of 39.3 % over the study period, Dake [24] projected that 11.2 %, 13.1 % and 15.1 % of Ghanaian women aged 15–49 years will be obese by 2013, 2018 and 2023, respectively.

Although the inexorable rise and public health consequences of overweight and obesity are evident in Ghana and in many African countries, our understanding of the key factors as well as the most at-risk groups for tailored interventions has advanced rather slowly. The current study, therefore, sought to compare temporal trends in overweight and obesity, and associated factors between parous and nulliparous women in Ghana. The study utilised high quality nationally representative data from the three most recent (2003, 2008 and 2014) Demographic and Health Surveys conducted in Ghana.

## Methods

### Data source

This study utilised data drawn from the 2003, 2008 and 2014 rounds of the Ghana Demographic and Health Surveys (GDHS), designed to provide data for monitoring the population and health situation in Ghana. The GDHS is a nationally representative survey of women and men aged 15–49 and 15–59, respectively. Similar to earlier GHDS, the 2003, 2008 and 2014 were based on a cross-sectional design in order to ensure that representative samples of the population were obtained. In all the surveys, a two-staged stratified sampling procedure was used, with the first stage involving the selection of clusters consisting of enumeration areas (EAs) using systematic random sampling based on probability proportional to population size. This was based on an updated master sampling frame constructed from the 2000 and 2010 Ghana Population and Housing Censuses, purposely to produce separate estimates for key indicators for each of the 10 regions of Ghana.

To ensure representativeness, the second stage involved a household listing operation in all selected clusters, followed by the systematic random sampling of the households listed in each cluster. In 2003, 2008 and 2014, a nationally representative sample of 5691, 4916, and 9396 women were interviewed, respectively. Similarly, 5015 (2003) 4568 (2008), and 4609 (2014) men were interviewed in the surveys. The response rates were generally high, ranging from 93.8 % in 2003 to 98.5 % in 2014. All individual respondents provided both written and verbal consent before each interview was conducted. Ethical clearance for the surveys was obtained from the Ghana Health Service Ethical Review Committee in Accra, Ghana.

The 2003, 2008 and 2014 surveys were chosen mainly because anthropometric data (height and weight) for all women aged 15–49 were measured. The earlier surveys (1988, 1993, and 1998) limited the collection of such data to only women who had a live birth in the 5 years (or 3 years) prior to the surveys. This excluded other women in their reproductive ages who had not yet initiated childbearing prior to the surveys [25]. The data currently used in this study, therefore, provide a more representative sample to facilitate comparability of trends and patterns between parous and nulliparous women. The three datasets used for the current study are publicly available and were requested online from http://dhsprogram.com/data/available-datasets.cfm .

### Analytical Sample

During the surveys, anthropometric data were measured for eligible women by trained personnel using standardised procedures. Details of the standardised procedures used for obtaining anthropometric measurements are available from the MEASURE DHS Biomarker Field Manual [26]. The analysis in this study was based on all women with complete data on height and weight for the purpose of calculation of body mass index (BMI). As typically done in obesity studies, pregnant women and lactating mothers were excluded from the study sample to avoid biased anthropometric estimates. The final sample for analysis was then stratified according to the two main groups of women (parous and nulliparous) in accordance with the purpose of the study. Hence, the analytical sample for the study consisted of 4834 (parous women, 3271; nulliparous women, 1563), 4380 (parous women, 2859; nulliparous women, 1521) 4268 (parous women, 2898; nulliparous women, 1370) women from the 2003, 2008 and 2014 GDHS, respectively.

### Dependent Variable

The dependent variable for the study was derived from BMI data of women in the surveys. Defined as weight in kilograms divided by the square of height in meters, the BMI is used to measure nutritional status based on standard World Health Organisation (WHO) [27] cut offs, namely; underweight, BMI < 18.5 kg/m^2^, normal weight, BMI of 18.5–25 kg/m^2^, overweight, BMI of 25.0–29.9 kg/m^2^; and obese BMI ≥ 30.0 kg/m^2^. The BMI method is an internationally recognized and a widely used method for defining overweight and obesity at the population level.

A BMI threshold of at least 25 kg/m^2^ has been identified to be associated with an elevated risk of mortality and non-communicable diseases [1, 27]. In view of this, a binary outcome was constructed as the dependent variable for the study, based on the WHO standard BMI cut offs [1, 27]. Thus, women with a BMI of 25.0 or above were considered as overweight/obese and coded “1”, while those below a BMI of 25.0 were considered otherwise and coded “0”. This categorisation was also done to ensure enough cases for analyses and to obtain more robust regression estimates [28].

### Independent Variables

Background characteristics of the women were used as independent variables for this study. Where necessary, some of the independent variables were transformed or recoded in order to make the analysis manageable and to facilitate comparison across the surveys and women groups. In that regard, age was grouped using a 10 year interval: 15–24, 25–34, 40–44, and 45+. Educational level was based on the highest level completed by each respondent and was grouped into four categories: no education, primary, middle/junior secondary school (JSS)/junior high school (JHS) and secondary/higher education. Four categories were created for marital status: never married, married, cohabiting, and previously married (divorced/widowed/separated). Occupational status was grouped thematically into five categories: not working, professional/managerial, sales/trade, agricultural and manual labour.

The DHS wealth quintile approach for measuring household wealth was adopted as a proxy for measuring wealth status: poorest, poorer, middle, rich and richest. With regards to ethnicity, the five major ethnic groups (Akan, Ga/Adangme, Ewe, Mole-Dagbani and Gruma) in Ghana were maintained, while minority groups including Guans, Mande, Grussi and other unspecified groups were categorised as “Others”. To take into account the effect of urbanization, type of locality was categorized into rural and urban. For parous women, the number of children (1, 2, 3, 4, 5, 6, 7+) they had given birth to was also included. A variable on survey year (2003, 2008, and 2014) was created from the three surveys.

### Analytical Strategies

In analysing data collected using complex survey designs such as DHS data, it is always important to take into consideration the survey design so as to obtain unbiased point estimates and accurate confidence intervals [29, 30]. With an in-built feature for estimating accurate standard errors in instances where the sample was drawn using clusters, stratification and unequal weights, STATA 11.0 software was deemed appropriate and used for data analyses in this study. In order to ensure representativeness and to correct for non-response, all the data used in this study were weighted. The complex survey design was also taken into consideration in the analyses, using the SVY command in Stata.

Given that the study was conceptualised on the premise of potential differences between parous women, and nulliparous women, all analyses were stratified according to the two women groups. Descriptive and inferential statistics were employed in this study. Descriptive statistics were used to analyse and present results of all variables considered in the study, and their relationship with overweight/obesity. In addition, a graphical representation of the trend in overweight/obesity according to the women groups over the survey periods was conducted.

In order to estimate the nature, strength and direction of association between the independent variables (socio-demographic factors) of interest and the dependent variable (overweight/obesity), it was necessary to conduct multivariate analyses. At this stage of the analyses, the three surveys were pooled given that a similar survey protocol in terms of design, scope, coverage, sampling, data collection, coding and weighting employed across the surveys. This also allowed for simplicity of reporting estimates while improving the statistical power of the analyses.

Binary logistic regression was the main statistical tool used to estimate the nature and strength of associations between women’s socio-demographic characteristics and overweight/obesity. Binary logistic regression was used because the dependent variable was constructed as a binary outcome. The binary logistic regression analyses were conducted both at the bivariate (Model I) and multivariate (Model II) levels for each women group (Table 3). This was aimed at exploring both the individual and joint effects of the independent variables on overweight/obesity. In the bivariate analyses, the independent effect of each of the explanatory variables (age, educational level, marital status, wealth quintile, occupation, type of locality, ethnicity, number of children (only parous women), and survey year) were assessed (Model I), while the multivariate analyses (Model II) estimated the joint effect of these explanatory variables.

To control for the effect of reproductive history (weight gain and retention associated with childbirth), number of children was included as a covariate in the model for parous women. The results of the logistic regression analyses were presented using odds ratios (OR) at 95 % confidence intervals (CIs). All analyses took into account clustering at the primary sampling level and weighting was factors to generate representative results. The Hosmer-Lemeshow goodness-of-fit test indicated that the models fit the data well.

## Results

### Socio-demographic characteristics of respondents

The socio-demographic characteristics of respondents for each survey according to the two women groups (parous and nulliparous) are presented in Table 1. In each of the surveys, most parous women were older than 25 years, whereas most nulliparous women were younger than 25 years. In 2014 for instance, about 86 % of parous women were above 25 years while about 79 % of nulliparous women were below 25 years in the same year. In both groups of women across the survey years, most respondents had attained middle/JSS/JHS education with the exception of parous women in 2003. In each survey, most parous women were married (2003, 74.3 %; 2008, 63.7 %; 2014, 56.8 %), while majority of nulliparous women were never married (2003, 86.0 %; 2003, 88.2 %; 2014, 88.7 %).

**Table 1:** Socio-demographic characteristics of women with BMI information by women groups: 2003, 2008 and 2014

	Parous women						Nulliparous women					
	2003		2008		2014		2003		2008		2014	
Characteristic	%	N	%	N	%	N	%	N	%	N	%	N
Age group (years)												
15–24	15.9	519	15.2	434	14.0	406	84.0	1313	82.7	1258	79.3	1086
25–34	38.0	1244	36.8	1053	36.2	1049	12.5	195	14.0	212	17.2	236
35–44	32.9	1076	33.9	969	35.7	1033	3.1	49	3.0	46	3.3	45
45+	13.2	432	14.1	403	14.1	410	0.4	6	0.3	5	0.2	3
Educational level												
No education	35.6	1164	28.5	815	26.0	753	11.4	179	5.6	85	3.9	54
Primary	6.7	219	23.2	662	19.9	577	14.2	222	14.1	215	14.7	201
Middle/JSS/JHS	25.4	832	46.1	1319	40.8	1183	56.6	885	73.7	1121	41.6	570
Secondary/higher	32.3	1056	2.2	63	13.3	385	17.8	277	6.6	100	39.8	545
Marital status												
Never married	3.7	121	6.2	178	8.2	237	86.0	1344	88.1	1341	88.7	1215
Married	74.3	2429	63.7	1821	56.8	1647	6.6	103	5.4	82	6.1	84
Cohabiting	8.7	286	16.0	458	18.9	549	4.4	69	4.6	69	3.9	54
Previously married	13.3	435	14.1	402	16.1	465	3.0	47	1.9	29	1.3	17
Wealth quintile												
Poorest	19.8	648	18.2	520	17.4	505	9.3	146	9.7	148	14.8	203
Poorer	19.3	630	20.3	580	18.0	521	11.2	176	12.9	196	15.0	206
Middle	20.6	672	20.4	583	22.3	647	15.0	234	19.2	292	17.6	241
Richer	19.9	652	22.8	652	22.0	638	26.2	409	24.9	379	23.1	316
Richest	20.4	669	18.3	524	20.3	587	38.3	598	33.3	506	29.5	404
Occupation												
Not working	9.5	311	8.8	252	13.0	380	51.6	806	52.1	792	45.4	622
Prof/managerial	8.5	278	11.5	330	6.8	197	13.5	211	18.2	277	12.5	171
Sales/trade	30.6	1002	39.7	1136	42.9	1240	16.0	250	14.4	219	23.9	328
Agric	37.4	1224	32.0	910	23.9	691	7.2	113	6.6	101	7.3	100
Manual	14.0	456	8.0	231	13.4	390	11.7	183	8.7	132	10.9	149
Type of locality												
Urban	41.7	1365	43.9	1255	51.7	1498	64.8	1013	59.5	905	61.4	841
Rural	58.3	1906	56.1	1604	48.3	1400	35.2	550	40.5	616	38.6	529
Ethnicity												
Akan	50.0	1634	50.5	1446	51.7	1499	53.6	838	52.9	806	50.4	690
Ga/Adangme	8.7	285	6.4	182	7.3	211	8.8	137	8.7	132	8.8	121
Ewe	13.3	437	13.0	372	12.9	373	14.1	220	12.1	184	13.7	188
Mole-Dagbani	13.1	429	17.0	485	14.1	410	8.8	137	12.8	195	13.9	191
Gurma	2.5	82	3.6	103	5.8	168	1.7	27	3.2	48	5.5	75
Others	12.4	404	9.5	271	8.2	237	13.0	204	10.3	156	7.7	105
Number of children												
1	20.1	658	19.8	565	19.3	558	-	-	-	-	-	-
2	16.9	553	19.4	556	20.1	582	-	-	-	-	-	-
3	16.3	532	16.7	477	17.4	506	-	-	-	-	-	-
4	12.9	424	15.1	431	14.1	409	-	-	-	-	-	-
5	10.8	351	10.4	296	11.2	325	-	-	-	-	-	-
6	8.8	288	7.5	213	8.3	239	-	-	-	-	-	-
7+	14.2	465	11.2	321	9.6	279	-	-	-	-	-	-
BMI group												
Underweight	8.0	262	6.8	193	4.1	119	12.0	187	12.1	185	10.6	145
Normal	61.9	2025	57.2	1636	48.0	1392	72.8	1138	69.4	1055	65.8	902
Overweight	19.6	640	23.9	685	28.3	820	12.3	192	14.5	221	17.4	239
Obese	10.5	344	12.1	345	19.6	567	2.9	46	4.0	60	6.2	84
Total	100	3271	100	2859	100	2898	100	1563	100	1521	100	1370

Whereas parous women seemed to be fairly distributed across the wealth quintiles in each survey, the percentage of nulliparous women seemed to increase with wealth quintiles in each survey. Apart from the 2003 survey, parous women were mostly engaged in sales/trade (2008, 39.7 %; 2014, 42.9 %) occupations. Most nulliparous women, on the other hand, were not working in all three surveys (2003, 51.6 %; 2008, 52.1 %; 2014, 45.4 %). Except for the latest survey (2014), more than half of all parous women resided in rural areas, in contrast to more than half of all nulliparous women resident in urban areas in all three surveys. The Akan ethnic group dominated both women groups in the three surveys.

Across the surveys, about a fifth of parous women had a child except for the most recent survey where this was the case for women with two children. With respect to BMI classification, the prevalence of underweight reduced with each subsequent survey for both women groups; ranging from 8.0 % in 2003 to 4.1 % in 2014 among parous women, and from 12.0 % in 2003 to 10.6 % in 2014 for nulliparous women. In contrast, the prevalence of both overweight and obesity increased with each subsequent survey for both women groups, with higher prevalence among parous compared with nulliprous women. This ranged from 19.6 % in 2003 to 28.3 % in 2014 for overweight and from 10.5 % in 2003 to 19.6 % in 2014 for obesity among parous women; while for nulliparous women, the prevalence ranged from 12.3 % in 2003 to 17.4 % in 2014 for overweight and from 2.9 % in 2003 to 6.2 % in 2014 for obesity.

### Prevalence of overweight/obesity by socio-demographic characteristics

**Fig. 1:**
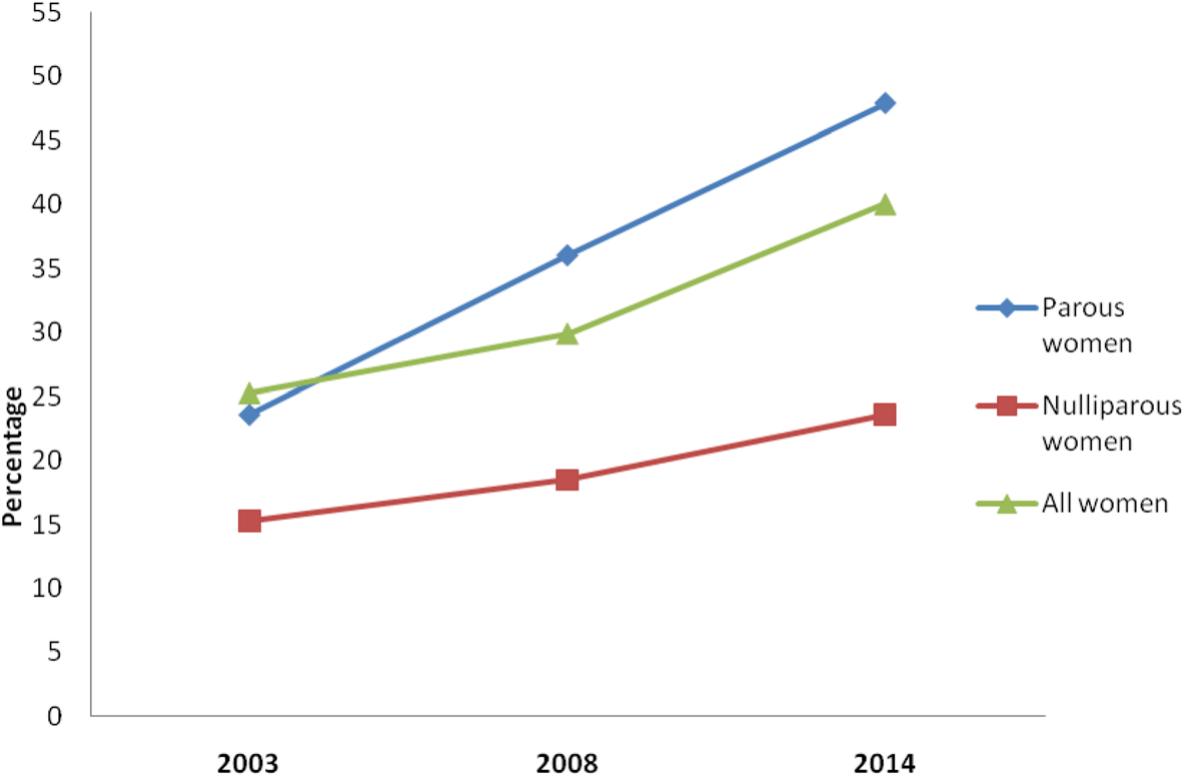
Prevalence of overweight/obesity among women (15–49 years) by year of survey

The prevalence of overweight/obesity in relation to socio-demographic characteristics of women is shown in Table 2. Generally, the prevalence of overweight/obesity increased linearly with each subsequent survey, with remarkable increases observed among parous women compared with their nulliparous counterparts. This ranged from about 30 % in 2003 to about 48 % in 2014 for parous women, compared with about 15 % to about 24 % for nulliparous women over the same period. These differences between the two women groups are clearly represented graphically in Fig. 1. With the exception of those aged 45 years and above, overweight/obesity increased with age among both women groups across the surveys with higher proportions observed among nulliparous women. For instance, in the most recent survey (2014), about 55 % of parous women aged 35–44 were overweight/obese compared with 73 % of nulliparous women in the same age group.

**Table 2:** Prevalence of overweight/obesity by socio-demographic characteristics of women: 2003, 2008 and 2014

	Parous women			Nulliparous women		
	2003 (*N* = 3271)	2008 (*N* = 2859)	2014 (*N* = 2898)	2003 (*N* = 1563)	2008 (*N* = 1521)	2014 (*N* = 1370)
Characteristic	%	%	%	%	%	%
Age group (years)						
15–24	12.4	13.2	24.4	11.9	14.2	15.2
25–34	29.1	36.6	47.9	28.3	36.2	52.0
35–44	38.1	43.6	55.1	51.5	48.7	73.5
45+	34.1	41	52.7	30.3	60.3	69.1
Educational level						
No education	18.0	21.9	27.5	11.8	14.8	18.4
Primary	35.0	35.3	47.8	13.0	14.3	8.5
Middle/JSS/JHS	31.5	43.4	54.8	15.5	17.9	17.0
Secondary/higher	41.3	72.3	66.6	18.3	36.8	36.7
Marital status						
Never married	12.8	29.3	33.4	12.9	16.4	19.7
Married	31.3	37.4	50.0	31.1	39.8	59.4
Cohabiting	25.3	27.5	40.8	21.3	28.4	39.9
Previously married	31.3	42.5	56.1	38.6	30.7	74.4
Wealth quintile						
Poorest	8.9	12.7	15.9	2.6	8.4	4.3
Poorer	15.6	18.5	31.3	2.7	7.8	7.6
Middle	23.0	30.3	45.5	5.3	9.1	21.1
Richer	38.2	50.9	63.9	16.3	24.5	29.1
Richest	63.5	66.4	75.3	25.1	26.4	38.6
Occupation						
Not working	27.7	33.4	37.6	11.4	10.7	15.3
Prof/managerial	48	46.4	67.9	22.5	32.5	47.5
Sales/trade	45.2	48.3	61.0	24.2	30.8	29.2
Agric	14.1	18.6	21.9	4.9	11.2	6.2
Manual labour	30.4	32.4	51.7	18.0	20.9	29.6
Type of locality						
Urban	46.8	52.4	60.0	19.3	23.5	29.2
Rural	18.1	23.3	34.9	7.7	11.1	14.6
Ethnicity						
Akan	33.2	40.9	55.9	15.4	17.7	26.4
Ga/Adangme	47.1	43.6	58.4	18.5	32.8	32.8
Ewe	31.7	40.4	44.8	14.6	15.0	26.3
Mole-Dagbani	13.2	22.9	27.8	11.1	12.1	13.6
Gurma	6.2	5.7	18.2	12.3	10.1	10.1
Others	26.7	33.6	48.5	16.0	25.0	17.6
Number of children						
1	23.7	30.1	40.7	-	-	-
2	36.9	39.2	54.8	-	-	-
3	32.3	36.9	54.4	-	-	-
4	35.6	40.7	50.7	-	-	-
5	31.1	39.8	42.8	-	-	-
6	24.0	33.0	50.0	-	-	-
7+	26.5	31.8	35.8	-	-	-
Total	30.1	36.0	47.9	15.2	18.5	23.6

Across the surveys, overweight/obesity was more common among those with secondary/higher education. However, parous women (2003, 41.3 %; 2008, 72.3 %; 2014, 66.7 %) were more affected compared with nulliparous women (2003, 18.3 %; 2008, 36.8 %; 2014, 36.7 %). With each subsequent survey, overweight/obesity increasingly affected parous women who had previously married (2003 31.3 %; 2008, 42.5 %; 2014, 56.1 %) more than the other categories of marital status. But for 2008, the trend for nulliparous women with respect to marital status was similar to that of parous women. In terms of wealth, the prevalence of overweight/obesity increased with increasing wealth quintile and survey, with the highest prevalence observed among parous women in the richest wealth quintile (75.3 %) in the 2014 survey.

The prevalence of overweight/obesity was higher in the urban than in the rural areas in both women groups, but increased with each subsequent survey. This ranged from about 47 % to 60 % and from about 19 % to 29 % among parous and nulliparous women, respectively. In terms of ethnicity, the Akans had higher rates of overweight/obesity in both women groups in all three surveys.

### Socio-demographic predictors of overweight/obesity

Table 3 shows the results of multivariate logistic regression examining the effects of socio-demographic characteristics of parous and nulliparous women on overweight/obesity based on pooled data from the three surveys (2003, 2008 and 2014) considered in this study. The aim was to examine how factors associated with overweight/obesity operate similarly or dissimilarly with respect to the two women groups. Number of children was included in the regression analyses for parous women to ascertain the effect of reproductive history on overweight/obesity among women who had previously given birth.

**Table 3:** Logistic regression results on socio-demographic predictors of overweight/obesity among women in Ghana, GHDS: 2003–2014

	Parous		Nulliparous	
Characteristic	Model I OR [95 % CI]	Model II OR [95 % CI]	Model I OR [95 % CI]	Model II OR [95 % CI]
Number of children				
1	1.0	1.0	na	na
2	1.732**	1.369**	na	na
	[1.473,2.036]	[1.123,1.670]	na	na
3	1.548**	1.114	na	na
	[1.310,1.831]	[0.896,1.385]	na	na
4	1.624**	1.179	na	na
	[1.366,1.931]	[0.934,1.487]	na	na
5	1.339**	1.100	na	na
	[1.111,1.615]	[0.857,1.412]	na	na
6	1.192	1.309	na	na
	[0.973,1.462]	[0.997,1.720]	na	na
7+	0.975	1.348	na	na
	[0.809,1.174]	[1.034,1.758]	na	na
Age group (years)				
15–24	1.0	1.0	1.0	1.0
25–34	3.072**	2.404**	4.138**	2.031**
	[2.561,3.685]	[1.928,2.997]	[3.319,5.159]	[1.562,2.641]
35–44	4.300**	3.528**	8.623**	3.584**
	[3.587,5.156]	[2.760,4.510]	[5.616,13.24]	[2.152,5.967]
45+	3.799**	3.314**	5.905**	3.205*
	[3.089,4.673]	[2.506,4.381]	[1.898,18.37]	[1.005,10.22]
Educational level				
No education	1.0	1.0	1.0	1.0
Primary	2.415**	1.517**	0.858	0.885
	[2.070,2.817]	[1.257,1.831]	[0.558,1.318]	[0.561,1.397]
Middle/JSS/JHS	2.881**	1.296**	1.279	0.932
	[2.540,3.268]	[1.095,1.534]	[0.893,1.831]	[0.629,1.381]
Secondary/higher	3.461**	1.467**	2.848**	1.171
	[2.973,4.029]	[1.201,1.793]	[1.946,4.167]	[0.764,1.796]
Marital status				
Never married	1.0	1.0	1.0	1.0
Married	1.658**	1.496**	3.835**	1.789**
	[1.314,2.091]	[1.115,2.008]	[2.801,5.252]	[1.208,2.649]
Cohabiting	1.289	1.127	2.120**	1.510
	[0.990,1.679]	[0.825,1.539]	[1.459,3.080]	[0.996,2.289]
Previously married	2.058**	1.428*	3.883**	2.161*
	[1.591,2.663]	[1.040,1.961]	[2.298,6.559]	[1.190,3.925]
Wealth quintile				
Poorest	1.0	1.0	1.0	1.0
Poorer	1.949**	1.503**	1.245	1.126
	[1.611,2.358]	[1.220,1.852]	[0.735,2.107]	[0.651,1.947]
Middle	3.533**	2.220**	2.516**	2.238**
	[2.952,4.227]	[1.786,2.759]	[1.588,3.986]	[1.341,3.733]
Richer	7.472**	4.132**	5.588**	4.480**
	[6.253,8.927]	[3.241,5.268]	[3.663,8.526]	[2.678,7.493]
Richest	15.49**	7.648**	7.792**	5.825**
	[12.86,18.65]	[5.836,10.02]	[5.162,11.76]	[3.438,9.869]
Occupation				
Not working	1.0	1.0	1.0	1.0
Prof/managerial	2.197**	1.315*	3.568**	1.800**
	[1.760,2.743]	[1.023,1.689]	[2.799,4.548]	[1.361,2.381]
Sales/Trade	2.184**	1.435**	2.801**	1.553**
	[1.835,2.599]	[1.178,1.749]	[2.216,3.542]	[1.185,2.035]
Agriculture	0.426**	0.702**	0.567*	0.953
	[0.354,0.514]	[0.561,0.879]	[0.357,0.903]	[0.581,1.562]
Manual labour	1.261*	1.086	2.090**	1.360
	[1.022,1.556]	[0.857,1.377]	[1.557,2.806]	[0.988,1.872]
Type of locality				
Urban	1.0	1.0	1.0	1.0
Rural	0.285**	0.958	0.402**	1.158*
	[0.257,0.316]	[0.825,1.113]	[0.330,0.489]	[0.892,1.504]
Ethnicity				
Akan	1.0	1.0	1.0	1.0
Ga/Adangbe	1.305**	1.195	1.591**	1.582**
	[1.081,1.576]	[0.957,1.493]	[1.191,2.127]	[1.151,2.173]
Ewe	0.831*	1.034	0.935	1.059
	[0.716,0.963]	[0.873,1.225]	[0.708,1.233]	[0.787,1.424]
Mole-Dagbani	0.357**	0.663**	0.584**	1.000
	[0.307,0.416]	[0.546,0.804]	[0.438,0.779]	[0.726,1.377]
Gurma	0.177**	0.495**	0.485*	0.947
	[0.124,0.251]	[0.345,0.709]	[0.266,0.884]	[0.479,1.871]
Others	0.694**	1.041	0.994	1.394*
	[0.590,0.817]	[0.860,1.260]	[0.752,1.313]	[1.025,1.897]
Survey year				
2003	1.0	1.0	1.0	1.0
2008	1.309**	1.389**	1.261*	1.404**
	[1.164,1.471]	[1.197,1.613]	[1.023,1.554]	[1.113,1.772]
2014	2.134**	2.386**	1.719**	1.888**
	[1.891,2.407]	[2.051,2.775]	[1.380,2.142]	[1.479,2.410]
*N*	9028	9028	4454	4454
Pseudo R^2^		0.1994		0.1468
Wald *X* ^2^ (df)		1401.38 (31)		403.06 (25)
Prob. > *X* ^2^		0.0000		0.0000
Hosmer-Lemeshow *X* ^2^ (df)		5.50 (8)		12.66 (8)
Prob. > *X* ^2^		0.7032		0.1242

*OR* Odds Ratios; 95 % confidence intervals in brackets, *p < .05, **p < .01; na = not applicable

Model I = Bivariate logistic regression analysis

Model II = Multivariate logistic regression analysis (age, educational level, marital status, wealth quintile, occupation, type of locality, ethnicity, number of children (only parous women), and survey year)

Model I (Table 3) shows the bivariate association between overweight/obesity and socio-demographic characteristics of women in each group (parous and nulliparous). For parous women, a significant positive association was found between overweight/obesity and women having children up to five (5) compared to those with one (1) child. With the exception of educational level and ethnicity, all the socio-demographic factors were significant and positively associated with overweight/obesity, although the odds varied between the women groups. While the odds of being overweight/obese were higher among nulliparous than parous women across all categories of age, marital status, occupation and type of locality, the reverse was the case for wealth status.

With reference to those with no education, the odds of being overweight/obese significantly increased with level of education (primary, OR = 2.45, 95 % CI = 2.070,2.817; middle/JSS/JHS, OR = 2.9, 95 % CI = 2.540,3.268; secondary/higher, OR = 3.5, 95 % CI = 2.973,4.029) for parous women, while only those with secondary/higher (OR = 2.8, 95 % CI = 1.946,4.167) education had significantly higher odds of being overweight/obese for nulliparous women. In terms of ethnicity, the Ga/Adangbe were significantly more likely to be overweight/obese for both parous (OR = 1.3, 95 % CI = 1.081, 1.576) and nulliparous (OR = 1.6, 95 % CI = 1.191, 2.127) women compared with Akans. In contrast, all the other ethnic groups were significantly less likely to be overweight/obese for parous women, while this was the case for nulliparious women of Mole-Dagbani and Gurma ethnicities.

In the multivariate analyses as shown in Model II for each women group, the magnitude of association between overweight/obesity and socio-demographic factors attenuated, but the direction of association and statistical significance remained unchanged, with a few exceptions. Unlike in the bivariate analyses (Model I), the multivariate analyses showed that parous women with two (2) children had significant higher odds (OR = 1.4, 95 % CI = 1.123, 1.670) of being overweight/obese compared with those with one (1) child.

Nonetheless, the results indicate a similar pattern of significant positive associations between overweight/obesity and age, marital and wealth quintile across both women groups, with variations in the magnitude of effect. Across all categories considered, age was consistently associated with overweight/obesity for both women groups. For instance, with reference to those aged 15–24 years, the odds of being overweight/obese were more than three times higher for both parous (OR = 3.5, 95 % CI = 2.760,4.510) and nulliparous (OR = 3.5, 95 % CI = 2.152,5.967) women aged 35–44 years. With respect to marital status, the positive significant effects of marital status on overweight/obesity were found to be stronger among nulliparous women (married, OR = 1.8, 95 % CI = 1.208, 2.649; previously married, OR = 2.2, 95 % CI = 1.190, 3.925), compared with their parous counterparts (married, OR = 1.4, 95 % CI = 1.115, 2.008; previously married OR = 1.4, 95 % CI = 1.040, 1.961).

In a similar way, the odds of being overweight/obese generally increased with wealth quintile for both women groups. For example, compared with those in the poorest quintile, the odds of being overweight/obese ranged from about 1.5 (95 % CI = 1.220, 1.852) in the poorer quintile to about 7.6 (95 % CI = 5.836, 10.02) in the richest quintile among parous women in 2014. For nulliparous women, on the other hand, the odds ranged from about 1.1 (95 % CI = 0.651, 1.947) to 5.8 (95 % CI = 3.438, 9.869) among those in the poorer and richest quintiles, respectively. However, the association between nulliparous women in the poorer wealth quintile was not statistically significant. Although the magnitude of effect reduced, the association between educational level and overweight/obesity remained significant in the multivariate analysis for parous women. In sharp contrast, none of the categories of educational level was found to be significantly associated with overweight/obesity for nulliparous women in model II.

Whereas all the categories of occupation were significantly associated with overweight/obesity at the bivariate level (Model I) for both women groups, this was not observed in the multivariate analyses (Model II). With reference to those not working, women in either group who engaged in professional/managerial (parous, OR = 1.3, 95 % CI = 1.023, 1.689; nulliparous, OR = 1.8, 95 % CI = 1.361, 2.381) and sales/trade (parous, OR = 1.4, 95 % CI = 1.178, 1.749; nulliparous, OR = 1.6, 95 % CI = 1.185, 2.035) occupations were significantly more likely to be overweight/obese. Although agriculture was the only category of occupation negatively associated with overweight/obesity, this was significant among parous (OR = 0.7, 95 % CI = 0.561, 0.879) women, but not their nulliparous counterparts in the multivariate analyses. In terms of type of locality (rural/urban), the significant negative effect of rural residence on overweight/obesity among parous women in the bivariate analyses (OR = 0.3, 95 % CI = 0.257, 0.316) became insignificant at the multivariate level. In contrast, the significant negative effect of rural residence on overweight/obesity among nulliparous women in the bivariate analyses (OR = 0.4, 95 % CI = 0.330, 0.489) became positive at the multivariate level of analyses (OR = 1.2, 95 % CI = 0.892, 1.504).

The effect of ethnicity on overweight/obesity widely varied between the two women groups in the multivariate analyses. With reference to the Akan ethnic group, parous women belonging to the Mole-Dagbani (OR = 0.7, 95 % CI = 0.546,0.804) and Gurma (OR = 0.5, 95 % CI = 0.345,0.709) ethnicities were significantly less likely to be overweight/obese, while the reverse was the case for their nulliparous counterparts belonging to the Ga/Adangbe (OR = 1.6, 95 % CI = 1.151,2.173) and those classified as “others” (OR = 1.4, 95 % CI = 1.025,1.897). As compared to the 2003 survey, the odds of being overweight/obese significantly increased with each subsequent survey, with stronger effects among parous than nulliparous women. For instance, in the most recent survey (2014), the odds of being overweight/obese were about 2.4 (95 % CI = 2.051, 2.775) times higher for parous women compared with about 1.9 (95 % CI = 1.479, 2.410) times higher for nulliparous women.

## Discussion

This is by far the first study to compare trends and socio-demographic factors associated overweight/obesity between parous and nulliparous women in Ghana using nationally representative samples. The prevalence of overweight/obesity consistently increased remarkably between 2003 and 2014 among the two women groups, with disproportionately higher rates among parous women compared with nulliparous women. The rates for parous women were about twice the rates of their nulliparous counterparts in each survey. Generally, this trend is consistent with prior studies on the subject in Ghana [24, 31], as well as current trends in most developing countries, and across the globe [1, 32]. This trend could be explained by substantial changes in lifestyle characterised by increased consumption of energy dense foods and reduced physical activity patterns. These changes currently being experienced in Ghana and many African countries have been linked with rapid economic growth, urbanisation, modernisation and globalisation of the food market [4, 33].

Further, the disproportionately higher rates found among parous women compared with nulliparous women could be attributed to weight gain and weight retention associated with pregnancy and childbirth [9, 11]. This could also be linked with pregnancy and postpartum related obesogenic cultures in Ghana which promote the consumption of so-called milk-inducing foods such as palm-nut soup which are fattening. Customary kin support and care for new mothers over the postpartum period compound the risk of overweight/obesity among parous women because of drastically reduced physical activity levels during this period [31, 34]. There is also a cultural dimension whereby kin members pressurise new mothers to eat in order to attain body weight considered to be symbolic of motherhood [35].

As premised in this study, disparities and consistencies were also found between parous and nulliparous with regards to socio-demographic factors associated with overweight/obesity. Overweight/obesity was consistently found to be significantly associated with increasing age across both women groups. Much as this finding mimics the expected body weight changes people undergo throughout the aging process, the finding also corroborates with prior studies conducted in Ghana [21, 31]. Similar to previous studies [31, 36], the current study found a direct positive association between wealth quintile and overweight/obesity, across both women groups. Wealth provides a means for individuals to seek the ideal body size expectations of their society. In developing countries, proof of wealth or success is often associated with a large body size [4, 37, 38], which could explain the association found between wealth and overweight/obesity in this study. This finding is, however, in contrast to studies in more developed countries which have reported inverse relation between wealth and overweight/obesity [16, 17].

Marital status may be associated with overweight and obesity either through the selection of individuals into and out of marital states, or through social causation where particular marital states influence weight gain [14, 39]. In many African cultures, both the selection and social causation hypotheses tend to be at play. A large sized woman is perceived as beautiful, healthy and more prestigious, thereby, increasing her chances of being selected into the state of marriage [40–42]. Nonetheless, there is some evidence of a gradual decline in the contribution of the historic Ghanaian socio-cultural valorisation of large-bodied women to the prevalence of overweight/obesity on one hand [4, 37], and an increasing contribution of lifestyles associated with urbanisation and westernisation [3, 5]. On the other hand, changes in social obligations, roles, and expectations associated with marriage often lead to increased food consumption and decreased time for physical activity [14]. These hypotheses seem to support the positive association found between marital status and overweight/obesity in both women groups.

Prior research in developing countries [17, 42] including Ghana [28, 31, 36] indicate that more educated people tend to have higher probability of being overweight/obese, partly linked with similar effects associated with socio-economic status. The findings of this study, however, suggest that the positive effect of education on overweight/obesity may be peculiar to parous women rather than the general population of women. Perhaps this is one highlight of the current study that needs to be explored further.

Depending on the level of work-related physical activity, occupation could either be a risk or protective factor associated with overweight and obesity. Particularly in developing countries such as Ghana, low-status jobs usually involve more physical activities and are protective against overweight and obesity than high-status jobs which usually involve relatively low physical exertion [43]. As expected, in this study, women engaged in agricultural occupations were less likely to be overweight/obese, but this was found only significant among parous women. On the other hand, overweight/obesity was found to be significantly more likely for both parous and nulliparous women in professional/managerial and sales/trade occupations.

Although the findings on occupation are consistent with Abdulai [21], the results on the relationship between occupation and overweight/obesity could be a reflection of broader structural changes on going in Ghana. As societies shift from being based on primary to secondary to tertiary production with increasing levels of incomes and industrialisation, more and more people shift from occupations that require high amounts of physical labour such as agriculture to those that require less physical labour [44]. Ghana has moved from a low income to a middle income country within the last decade, which could probably explain the disparities in overweight/obesity between the various occupations. Again, the results in Table 1 show increases in the proportion of women in professional/managerial and sales/trade occupations, and decreases in the proportion of women in agricultural occupations between 2003 and 2008, lending credence to this fact.

The positive effect of rural residence on overweight/obesity among nulliparous women contradicts previous reports that residing in a rural locality reduces one’s risk of being overweight/obese [28, 36, 45]. The sharp change in direction of effect from negative in the bivariate analyses to positive in the multivariate analyses, however, suggests that other factors may be involved. Perhaps, rural residents are gradually being exposed to similar risks of overweight/obesity as have previously been reported for urban residents. This could be explained by economic growth and associated pervasive diffusion of urban lifestyles to rural communities [46, 47].

Contrary to reports by prior studies in Ghana [31, 36, 48], the effect of ethnicity on overweight/obesity may not be as straightforward. For nulliparous women, the Ga/Adangbe and those classified as “Others” had higher probability of being overweight/obese. It is conceivable that nulliparous Ga/Adangbe women still hold on to the age-old socio-cultural perceptions that large-bodied women are attractive. As the single major ethnic group (Ga/Adangbe) in Accra, the Capital city of Ghana, the obesogenic lifestyles of the city may conveniently provide a means for women to attain such socio-cultural body image expectations.

In contrast, parous women of Mole-Dagbani and Gurma ethnicities had lower probability of being overweight/obese. This could be explained by the fact that these ethnic groups are found in the northern regions (Northern, Upper West and Upper East) of Ghana which are among the poorest in the country, and are predominantly rural farming communities [49]. The increasing odds of overweight/obesity with each subsequent survey could be an indication of the sweeping effects of rapid urbanisation across Ghana in recent times, coupled with the pervasive westernisation of diets and reduced physical activity patterns [34].

The strengths of this study stem from the fact that it utilised data from three large nationally representative surveys with high response rates. Thus, the results are to a large extent generalizable. To the best of our knowledge, this is the first study on the subject to stratify Ghanaian women in their reproductive ages (15–49 years) according to their reproductive history. The study, however, has some limitations that need to be acknowledged. Given the cross-sectional nature of the data used, inferences between socio-demographic characteristics of women and overweight/obesity could only be based on associations rather than causal links.

Further, as the variance associated with the multivariate results may suggest, other potential contributors to overweight/obesity such as genetics, dietary, and physical activity patterns could equally be important, but were not accounted for in the current analyses due to data constraints. Apart from BMI, the data did not also permit the inclusion of other measures of adiposity such as arm and waist circumference, and waist-to-hip ratio in our analyses. The results should therefore be interpreted bearing in mind these limitations. Nonetheless, the study extents the discourse on overweight and obesity in Ghana, and perhaps beyond.

## Conclusion

The prevalence of overweight/obesity among Ghanaian women in their reproductive ages (15–49 years) persistently increased between 2003 and 2014, but more sharply among parous women compared with nulliparous women. This trend warrants urgent public health attention considering that overweight/obesity increases risks for several non-communicable disease (such as diabetes, hypertension, cancers, heart disease), as well as adverse maternal (gestational diabetes, hypertension in pregnancy, pre-term delivery) and child health outcomes (congenital foetal anomalies, stillbirth).

Some socio-demographic factors (age, wealth, marital status) similarly predict overweight/obesity among both parous and nulliparous women. Yet again, other socio-demographic factors (education, type of locality, occupation and ethnicity) predict overweight/obesity differently between parous and nulliparous women. National level strategies aimed at preventing further increases in overweight and obesity should be tailored to account for the peculiar differences between parous and nulliparous women.

## Abbreviations

BMI: Body Mass Index
DHS: Demographic and Health Survey
GDHS: Ghana Demographic and Health Survey
JHS: Junior High School
JSS: Junior Secondary School
WHO: World Health Organization
